# Atypical Combination of Mixed Connective Tissue Disease and Multicentric Castleman Disease

**DOI:** 10.7759/cureus.70325

**Published:** 2024-09-27

**Authors:** Dosbai Saparov, Shakirat Gold-Olufadi, Mustafa Wasifusddin, Narek Hakobyan, Ifeanyi Uche, Henry Becerra, Philipp Barakat, Ruchi Yadav, Akriti Pokhrel, Avezbakiyev Boris, Jen Wang

**Affiliations:** 1 Internal Medicine, Brookdale University Hospital Medical Center, Brooklyn, USA; 2 Medicine, Brookdale University Hospital Medical Center, Brooklyn, USA; 3 Hematology and Medical Oncology, Brookdale University Hospital Medical Center, Brooklyn, USA; 4 Hematology and Oncology, Brookdale University Hospital Medical Center, Brooklyn, USA

**Keywords:** chemotherapy, leukemia, lymphoma, mixed connective tissue disease, multi-centric castleman’s disease

## Abstract

Castleman disease (CD) is a rare lymphoproliferative disorder with unicentric (UCD) and multicentric (MCD) forms, first detailed by Benjamin Castleman in 1956. It has three subtypes: hyaline vascular, plasma cell, and mixed. CD incidence is higher in HIV patients and is often associated with human herpes virus-8 (HHV-8). We report a 68-year-old woman with diabetes and mixed connective tissue disease (MCTD), which was diagnosed six months prior to presentation, who presented with lymphadenopathy, splenomegaly, and B symptoms. Imaging showed diffuse adenopathy. Biopsy confirmed the plasma cell subtype of MCD, with polyclonal plasmacytosis. The patient tested negative for HIV and HHV-8. Initial treatment with rituximab and corticosteroids resolved her symptoms. Six years later, she relapsed and was treated with an anti-IL-6 agent, which she could not complete due to adverse effects but still showed symptom improvement. This case is notable for the patient’s age and the plasma cell subtype of MCD, as well as the concurrent diagnosis of MCTD. The patient’s clinical presentation and histopathological findings underscore the importance of considering CD in the differential diagnosis of lymphadenopathy with systemic symptoms. Chronic inflammation and B lymphocyte proliferation, potentially linking MCTD and CD, were evident in this case. Despite extensive comorbidities, the patient remained clinically stable due to intensive multidisciplinary management. CD is a rare, heterogeneous disorder requiring a high index of suspicion. The potential link between CD and MCTD warrants further research. Effective management involves targeted therapies and close monitoring due to relapse risk. This case underscores the importance of individualized treatment plans considering comorbidities and treatment tolerability. Further research is needed to better understand CD's pathogenesis and develop effective treatments.

## Introduction

Castleman disease (CD) is a rare, non-clonal lymphoproliferative disorder first reported in 1954 and subsequently described in detail by Benjamin Castleman in 1956 when he reported a series of patients with localized mediastinal lymph node hyperplasia [1]. There are three main histological subtypes: hyaline vascular, plasma cell variant, and mixed variant [1]. Clinically, CD can be classified into the more localized unicentric (UCD) form or the generalized multicentric (MCD) form. The incidence of CD is increased in patients with human immunodeficiency virus (HIV). The human herpes virus-8 (HHV-8) is associated with almost all of the HIV-associated CD cases and approximately 50% of non-HIV cases [2]. Overproduction of interleukin-6 (IL-6) and the IL-6 receptors correlates with plasma cell CD. The proliferation of B lymphocytes and vasculogenesis leads to systemic non-specific clinical findings such as fever, anemia, hypoproteinemia, and proteinuria [3].

We present a case of a 68-year-old woman who tested negative for both HIV and HHV-8 and who exhibited clinical signs suggestive of MCD. She presented with a constellation of symptoms, including lymphadenopathy, splenomegaly, and B symptoms, necessitating a comprehensive differential diagnosis. Imaging, laboratory findings, and biopsy confirmed the diagnosis of the plasma cell subtype of MCD, a less common variant characterized by marked polyclonal plasmacytosis. This case is notable not only for the patient’s age and the subtype of CD but also for the concurrent diagnosis of mixed connective tissue disease (MCTD), suggesting a potential link between chronic inflammatory conditions and CD. The patient’s initial treatment with rituximab and systemic corticosteroids led to symptom resolution, highlighting the importance of targeted therapy in managing this rare disease. However, a relapse six years later required the introduction of anti-IL-6 therapy, which was only partially completed due to adverse effects but still resulted in significant symptom improvement.

## Case presentation

A 68-year-old female with a past medical history significant for diabetes mellitus (DM), hypertension (HTN), atrial fibrillation (AFib), and MCTD presented to the hospital on multiple occasions. Her symptoms included lymphadenopathy, splenomegaly, malaise, fever, and arthralgia predominantly affecting the small joints of the bilateral upper extremities, as well as night sweats. She had been on long-term low-dose prednisone therapy for her MCTD, which was diagnosed six months prior to presentation. Given the multisystem involvement and the patient's age, initial differential diagnoses included a broad spectrum of inflammatory, autoimmune, infectious, and malignant etiologies.

**Figure 1:**
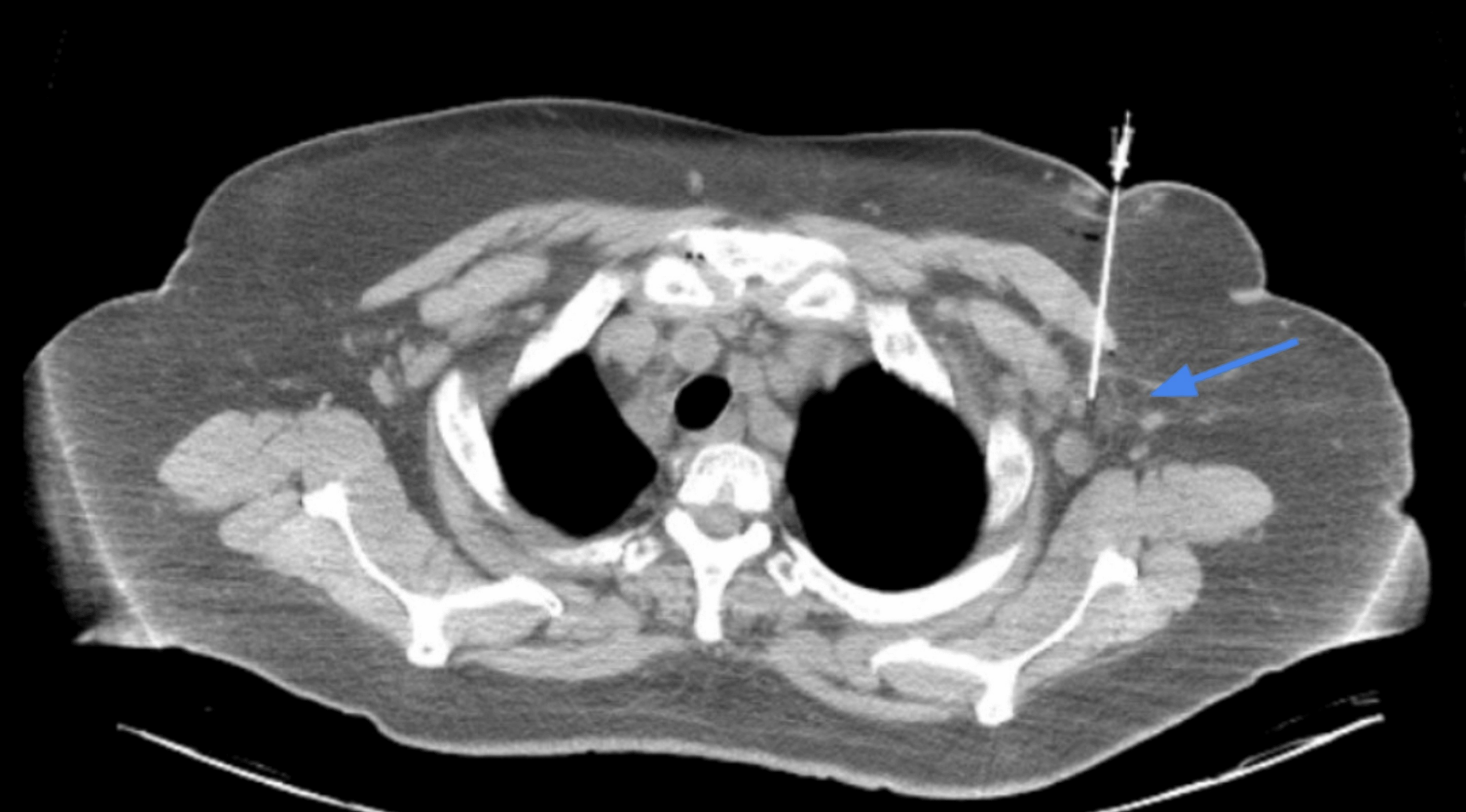
CT-guided core biopsy of a left axillary 2 cm lymph node (blue arrow).

Abdominal computed tomography (CT) scan revealed diffuse intra-abdominal adenopathy and chest CT showed 2 cm axillary adenopathy (Figure 1). CT-guided core biopsy (Figure 1) of the left axillary lymph node revealed follicular and paracortical hyperplasia with marked polyclonal plasmacytosis. Focal monocytoid B cell proliferation was observed within and around the open lymphoid sinuses, with rare germinal centers displaying regressive features but mostly appearing hyperplastic. Immunostaining demonstrated that CD20 and CD79a highlighted B cell areas, CD3 highlighted small lymphocytes in the paracortical area, and PD1 highlighted small T-cells within the follicles. CD25 marked scattered activated T cells. MUM-1, CD79a, kappa, and lambda marked increased polytypic plasma cells, while CD30 highlighted scattered immunoblast-like cells. CD21 and CD23 highlighted follicular dendritic meshworks confined to the follicles, and cyclin D1 showed some granulocytes. Ki-67 highlighted the germinal centers and lymphoid cells in the paracortical areas. Negative results were obtained for CD56, ALK-1, Epstein-Barr encoding region (EBER) in situ hybridization (ISH), and HHV-8, excluding the common association with Kaposi's sarcoma. Polymerase chain reaction (PCR) analysis revealed polyclonal T-cell receptor (TCR)-beta, TCR-gamma, IgH, and IgK gene rearrangement. The histologic features of angioimmunoblastic T-cell lymphoma were not observed. The patient was tested negative for HIV as well. Based on the clinical, imaging, laboratory, and histopathological findings, the patient was diagnosed with MCD, a rare lymphoproliferative disorder, specifically the less common plasma cell subtype of multicentric disease. Given the positive CD20 immunostaining, the patient was initially administered single-agent chemotherapy with rituximab, receiving weekly doses for the first four weeks, followed by monthly doses for four months, in conjunction with systemic corticosteroid therapy.

Furthermore, it is noteworthy that the patient received a diagnosis of MCTD approximately six months prior to the diagnosis of CD, indicating a potential association between these conditions through the pathways of chronic inflammation and B lymphocyte proliferation. Additionally, the differential diagnosis included POEMS syndrome (polyneuropathy, organomegaly, endocrinopathy, monoclonal plasma cell disorder, and skin changes) due to its established association with MCD; however, this diagnosis was not confirmed in the present case. Her condition was completely resolved with rituximab.

Six years later, the patient presented with similar symptoms, and her IL levels were found to be significantly elevated. This finding prompted the introduction of an anti-IL-6 agent. However, the patient was unable to tolerate this medication due to the onset of fatigue, arthralgias, nausea, and headache during the course of therapy, ultimately preventing her from completing the full treatment regimen. Despite the premature discontinuation of the anti-IL-6 agent midway through the course, the patient's symptoms showed improvement. Notwithstanding her extensive comorbidities, which include a history of AFib, DM, HTN, recurrent hospital admissions for disease flares, and interstitial lung disease (ILD) secondary to MCTD, she has remained clinically stable owing to intensive multidisciplinary management.

## Discussion

CD is a rare group of lymphoproliferative disorders that has a varied clinical presentation depending on the subtype [4]. The uncommon condition was first described in 1956 by Dr. Benjamin Castleman when he documented a case series of 13 patients with non-malignant mediastinal lymphadenopathy [5]. Clinically, it may present as the more common focal unicentric disease or the less commonly encountered multicentric disease with multi-systemic involvement [6]. It is often a great mimicker of inflammatory, autoimmune infectious, and malignant diseases with delayed diagnosis, especially when it presents as the multicentric type [7]. The etiology of CD is largely unknown but studies have revealed that it is a combination of immune dysregulation with resultant proliferation of B lymphocytes and plasma tissues in the lymphoid organs [8]. The inciting factor has been postulated to be related to elevated IL-6 [9], chronic inflammation, lymphoid expansion, chronic viral infections, especially HHV-8 infections, cytokine activation, and vascular proliferation [10]. HIV patients are at increased risk of the condition, which is strongly associated with HHV-8, hence Kaposi’s sarcoma is often a common co-existing condition [3]. The patient in this case was atypical, especially with regards to her age at presentation, and the clinical subtype she was diagnosed with. The frequently encountered subtype of CD in up to 90% of cases is the hyaline type, which tends to run a more benign course with most patients often diagnosed before the age of 30 [11]. Our patient presented with symptoms of multicentric disease such as lymphadenopathy and splenomegaly, and was both HIV and HHV-8 negative based on negative PCR and immunohistochemistry of blood and lymph node specimens. HHV-8 is associated with almost all cases of HIV+ MCD, while only 40-50% of HIV- MCD cases are associated with HHV-8 [5]. Chronic inflammation and proliferation of B lymphocytes are part of the postulated mechanisms involved in the etiology of CD and this may be the link between MCTD and CD in our patient. B symptoms of malaise, fever joint pains, and night sweats are often encountered in both conditions and the diagnosis of one of the conditions would have been easily missed without a heightened clinical acumen supported by focused investigations, including tissue biopsy with histological confirmation. The plasma cell type of MCD in histology is classically typified by infiltration by plasma cells in the mantle zone and hyperplastic germinal centers, which was seen in the histology of the lymph node biopsy in our patient [12]. Several conditions have been associated with MCD, including POEMS syndrome and autoimmune disorders [13-15].

CD20 was positive in the immunostaining, which informed the choice of rituximab as the initial treatment. The use of systemic corticosteroids may have also modified the outcome as the patient had systemic steroids before she commenced rituximab. IL-6 levels were high in our patient, which informed the use of an anti-IL-6 agent as a second-line treatment. Interestingly, anti-IL-6, which has proven very effective in managing MCD, was not tolerated by our patient because of significant adverse effects. Other medications used in managing MCD include chemotherapy such as cyclophosphamide, doxorubicin, vincristine, and prednisolone (CHOP) azathioprine with varying degrees of response [2,16]. There have been several articles of improvement in the clinical state and resolution of disease after treatment with anti-IL-6 medications, which still remain the first-line treatment for MCD [17]. Although MCD has a worse prognosis, our patient is still clinically stable despite the extensive co-morbidities, including a precarious cardiac history of AFib, HTN, DM, recurrent hospital admissions for flares, and ILD from MCTD, which is largely because of an intensive team effort. The importance of multidisciplinary management was also brought to the fore when managing this index patient and this was a crucial aspect of management.

## Conclusions

CD is a rare and heterogeneous group of lymphoproliferative disorders with diverse clinical presentations. The case presented here highlights the importance of a high index of suspicion, particularly in patients with multisystem involvement and non-specific symptoms. The potential association between CD and MCTD in this case provides a basis for further research into the underlying mechanisms of these conditions. It is essential to monitor patients closely, even after successful initial treatment, as relapses may occur. The choice of treatment should be individualized, considering the patient's comorbidities, subtype of CD, and tolerability to available therapeutic options. Although she could not complete the full course of anti-IL-6 therapy due to adverse effects, a partial course still led to significant symptom improvement, highlighting the potential effectiveness of even incomplete anti-IL-6 treatment. Further studies are needed to elucidate the pathogenesis of CD and to identify targeted therapies with better efficacy and tolerability.
